# Genetic diversity estimation of Yunnan indigenous goat breeds using microsatellite markers

**DOI:** 10.1002/ece3.5174

**Published:** 2019-04-29

**Authors:** Guang‐Xin E, Qiong‐Hua Hong, Yong‐Ju Zhao, Yue‐Hui Ma, Ming‐Xing Chu, Lan Zhu, Yong‐Fu Huang

**Affiliations:** ^1^ College of Animal Science and Technology, Chongqing Key Laboratory of Forage & Herbivore, Chongqing Engineering Research Centre for Herbivores Resource Protection and Utilization Southwest University Chongqing China; ^2^ State Key Laboratory of Genetic Resources and Evolution, Kunming Institute of Zoology Chinese Academy of Sciences Kunming China; ^3^ Yunnan Animal Science and Veterinary Institute Kunming China; ^4^ Institute of Animal Science Chinese Academy of Agricultural Sciences (CAAS) Beijing China

**Keywords:** China, diversity, indigenous goat, microsatellite, Yunnan

## Abstract

**Background:**

To assess the genetic diversity of seven Yunnan indigenous goat populations (Fengqing hornless goat, Mile red‐bone goat, Longling goat, Ninglang black goat, Black‐bone goat, Yunling black goat, and Zhaotong goat), their population structures were investigated using 20 microsatellite markers.

**Results:**

The results indicated that the genetic diversity of these goats was rich. The observed heterozygosity ranged from 0.4667 ± 0.0243 to 0.5793 ± 0.0230, and the mean number of alleles ranged from 4.80 ± 1.61 and 4.80 ± 1.64 to 6.20 ± 2.93. The population structure analysis showed that these seven goat populations were separated into two clusters, consistent with the results from phylogenetic networks, pairwise differences, and STRUCTURE analyses. We speculate that this may have been caused by natural geographical isolation, human migration and economic and cultural exchanges. We suggest removing CSRD247 and ILSTS005, two loci identified to be under positive selection in the present study, from the microsatellite evaluation system of goats.

**Conclusions:**

The present study may provide a scientific basis for the conservation and utilization of Yunnan indigenous goats.

## 1 BACKGROUND

Goats (*Capra hircus*) are economically important domestic animals worldwide. Currently, the abundant genetic diversity of goats has been assessed by using many different types of molecular markers, including microsatellites (Câmara, Nunes, Diniz, Silva, & Araújo, 2017; E et al., 2018), MHC region variants (Gowane, Akram, Misra, Prakash, & Kumar, 2018), mitochondrial DNA (E et al., 2018; Tarekegn et al., 2018), functional genes (Gowane et al., 2018; Pitarch et al., 2018), and genome‐wide SNPs (Ilie, Kusza, Sauer, & Gavojdian, 2018; Nicoloso et al., 2015; Onzima et al., 2018). The diversity of several indigenous goat breeds in China was assessed (E et al., 2018; Liu et al., 2014; Wang et al., 2017).

Yunnan is located in a low‐latitude inland area at the southwestern border of China with a subtropical plateau monsoon type climate (Liu, Wang, & Zhang, 2016). In addition, Yunnan Province contains the largest ethnic group in China, including 25 ethnic minorities, with populations over 6,000. Among these individuals, 15 ethnic groups are unique to Yunnan, and this population accounts for more than 80% of the total ethnic population of China (Pu, 2010). Furthermore, Yunnan is one of the important birthplaces of human civilization in China. The Yuanmou people, who lived 1.7 million years ago, were the earliest human beings in China and Asia (Gao, 2015; Yao, Deng, & Zhu, 2005). During the Warring States Period, the Yi tribe lived in Yunnan (Pu, 2010). Moreover, the number of plant and animal species in Yunnan is the highest in China.

Goats are the most important source of meat in this region and play an important role in economic development and social stability. Additionally, goats are also the objects of totem worship of many local minorities, affecting the spiritual worlds of the local indigenous peoples (Lai, 2012).

According to historical records, the domestication of local goats occurred 3,000 years ago (China National Commission of Animal Genetic Resources, 2011). Through long‐term natural selection and artificial selection, a large number of indigenous goat breeds with environmental adaptations and special genetic phenotypes have been created (Wang et al., 2017). For example, Ninglang black goat (NLG) and Yunling black goat (YLG) have black coats, Zhaotong goat (ZTG) and Longling goat (LHG) have extremely high slaughter rates, Mile red‐bone goat (HGG) and Black‐bone goat (WGG) have significant bone pigmentation, and Fengqing hornless goat (FQG) are naturally hornless or have horn dysplasia (China National Commission of Animal Genetic Resources, 2011).

Therefore, an assessment of the diversity and population structure of these goats not only helps to understand their domestic history and conservation status but also plays an important supporting role for future breeding improvement and industrial development of local goat populations.

## 2 METHODS

### 2.1 Animals, DNA extraction, and genotyping

The experimental conditions in the present study were approved by the Committee on the Ethics of Animal Experiments of Southwest University (No. [2007] 3) and the Animal Protection Law of China (this study did not directly carry out any relevant experiments on animals). All samples were collected from venous blood. The sample supply department guaranteed that after blood collection, the blood collection site was disinfected with alcohol to ensure no infection. At the same time, the animals were observed to survive normally after 1 week without any adverse effects. The genomic DNA from 175 individual blood samples of seven Yunnan indigenous goat populations (sample information see Table 1), which was supported by the Yunnan Animal Science and Veterinary Institute, was extracted by a standard phenol–chloroform protocol (Sambrook & Russell, 2001) and quantified with a DTX microplate reader (Beckman Coulter).

**Table 1:** Sample information for the seven Yunnan goat populations

Name	Style	Code	SZ	N	E	Native location
Fengqing hornless goat	Indigenous	FQG	23	24°35′0.72″	99°55′39.01″	Fengqing, Yunnan, China
Mile red‐bone goat	Indigenous	HGG	21	24°24′48.03″	103°24′46.39″	Mile, Yunnan, China
Longling goat	Indigenous	LHG	27	24°35′23.56″	98°41′20.07″	Longling, Yunnan, China
Ninglang black goat	Indigenous	NLG	28	27°17′7.87″	100°51′2.79″	Ninglang, Yunnan, China
Black‐bone goat	Indigenous	WGG	21	26°27′24.74″	99°24′55.75″	Lanping, Yunnan, China
Yunling black goat	Indigenous	YLG	31	25°43′18.52″	101°19′22.58″	Chuxiong, Yunnan, China
Zhaotong goat	Indigenous	ZTG	24	27°20′29.68″	103°42′52.88″	Zhaotong, Yunnan, China

Note

SZ is Sample size, N is North latitude, E is East longitude, and Code is short name of breed.

We used 20 microsatellites, which were recommended by ISAG‐FAO, to genotype the 175 individuals (FAO, 2011) (Table 2). Approximately 2 µl of PCR product was diluted with 10 µl of autoclaved distilled water for use in DNA genotyping. Two microliters of the diluted product were added to 7.75 µl of Hi Di™ formamide and 0.25 µl of GeneScan 500 LIZ dye size standard. The mixture was heated at 94°C for 5 min and then immediately chilled on ice for 2 min. Genotyping was performed using a Genetic Analyser 3130 xl (AB Applied Biosystems), according to E et al. (2018).

**Table 2:** Information for the 20 genomic microsatellite markers examined in the present study

Locus name	Primer sequence（5′–3′）	Tm (°C)	FL（bp）	LM
MAF065	F:AAAGGCCAGAGTATGCAATTAGGAG R:CCACTCCTCCTGAGAATATAACATG	58	116–158	FAM
MAF70	F:CACGGAGTCACAAAGAGTCAGACC R:GCAGGACTCTACGGGGCCTTTGC	65	134–168	FAM
SRCRSP23	F:TGAACGGGTAAAGATGTG R:TGTTTTTAATGGCTGAGTAG	58	81–119	HEX
OarFCB48	F:GAGTTAGTACAAGGATGACAAGAGGCAC R:GACTCTAGAGGATCGCAAAGAACCAG	58	149–173	FAM
SRCRSP9	F:AGAGGATCTGGAAATGGAATC R:GCACTCTTTTCAGCCCTAATG	58	99–135	FAM
OarAE54	F:TACTAAAGAAACATGAAGCTCCCA R:GGAAACATTTATTCTTATTCCTCAGTG	58	115–138	ROX
SRCRSP8	F:TGCGGTCTGGTTCTGATTTCAC R:GTTTCTTCCTGCATGAGAAAGTCGATGCTTAG	55	215–255	FAM
SPS113	F:CCTCCACACAGGCTTCTCTGACTT R:CCTAACTTGCTTGAGTTATTGCCC	58	134–158	HEX
OarFCB20	F:GGAAAACCCCCATATATACCTATAC R:AAATGTGTTTAAGATTCCATACATGTG	58	93–112	FAM
CSRD247	F:GGACTTGCCAGAACTCTGCAAT R:CACTGTGGTTTGTATTAGTCAGG	58	220–247	HEX
INRA063	F:GACCACAAAGGGATTTGCACAAGC R:AAACCACAGAAATGCTTGGAAG	58	164–186	FAM
ILSTS011	F:GCTTGCTACATGGAAAGTGC R:CTAAAATGCAGAGCCCTACC	58	250–300	FAM
ILSTS005	F:GGAAGCAATTGAAATCTATAGCC R:TGTTCTGTGAGTTTGTAAGC	55	172–218	HEX
SRCRSP15	F:CTTTACTTCTGACATGGTATTTCC R:TGCCACTCAATTTAGCAAGC	55	172–198	FAM
ILSTS029	F:TGTTTTGATGGAACACAG R:TGGATTTAGACCAGGGTTGG	55	148–170	FAM
TGLA53	F:GCTTTCAGAAATAGTTTGCATTCA R:ATCTTCACATGATATTACAGCAGA	55	126–160	HEX
MAF209	F:GATCACAAAAAGTTGGATACAACCGTG R:TCATGCACTTAAGTATGTAGGATGCTG	55	100–104	HEX
INRABERN185	F:CAATCTTGCTCCCACTATGC R:CTCCTAAAACACTCCCACACTA	55	261–289	HEX
TCRVB6	F:GAGTCCTCAGCAAGCAGGTC R:CCAGGAATTGGATCACACCT	55	217–255	HEX
SRCRSP7	F:TCTCAGCACCTTAATTGCTCT R:GGTCAACACTCCAATGGTGAG	55	117–131	FAM

Note

F, forward primer; FL, fragment length; LM, label marker; R, revise primer; Tm, annealing temperature.

### 2.2 Statistical analysis

Statistical analysis of the observed (*H*_o_) heterozygosity, expected heterozygosity (*H*_E_), mean number of alleles (*N*_A_), and polymorphism information content (PIC) was performed with the Microsatellite Toolkit (Park, 2008). Deviations from Hardy–Weinberg equilibrium (HWE) of the markers within the population were estimated using GENEPOP 3.4 software (Raymond & Rousset, 1995). The inbreeding coefficient (*F*_IS_) was calculated by Fisher's exact test with Bonferroni's correction using FSTAT 2.9.3.2 (Goudet, 1995). Pairwise differences between the populations (*F*_ST_) were assessed with Arlequin software version 3.5.1.3 (Excoffier & Lischer, 2010).

A phylogenetic neighbor‐joining network was reconstructed with Reynolds genetic distance (Reynolds, Weir, & Cockerham, 1983) by using the PHYLIP software package (Felsenstein, 2005) and visualized by SPLITSTREE4 (Kloepper & Huson, 2008).

STRUCTURE 2.3.3 was utilized to assess the population structure with Bayesian clustering and an admixture model from *K* = 2 to *K* = 11 in 100 runs. Assignment clusters were made with a burn‐in of 50,000, and 100,000 Markov Chain Monte Carlo iterations. CLUMPP software (Jakobsson & Rosenberg, 2007) was used to merge all runs for each *K*, and the results were visualized by DISTRUCT 1.1 (Rosenberg, 2004). Finally, the best *K* value was estimated using the STRUCTURE Harvester (Earl & vonHoldt, 2012) online tool.

## 3 RESULTS

Across breeds, an average of 11.4 alleles per marker was observed, ranging from 5 (ILSTS005) to 22 (SRCRSP23). The mean *H*_o_ and *H*_E_ within loci across the populations were 0.5209 (0.2508 [ILSTS029] to 0.7134 [SRCRSP8]) and 0.5994 (0.3387 [ILSTS029] to 0.7298 [SRCRSP9]), respectively. The average PIC across loci was 0.5436 and ranged from 0.3003 (ILSTS029) to 0.7680 (SRCRSP23) among all populations (Table 3).

**Table 3:** Genetic diversity statistics for each locus across all populations

Locus	Genetic diversity				dHWE	Locus under selection		
	*H*_o_	*H*_E_	PIC	*N*_A_		Obs. Het. BP	Obs* F*_ST_	*F*_ST_ *p*‐value
ILSTS005	0.3615	0.6378	0.5528	5	6	0.7983	0.2006	0.0043
INRABERN185	0.3047	0.3604	0.3259	7	0	0.3775	0.0501	0.1411
MAF065	0.6505	0.7295	0.6691	12	0	0.7816	0.0670	0.1906
INRA063	0.621	0.6708	0.6091	7	0	0.7337	0.0807	0.3825
ILSTS011	0.5352	0.5593	0.5052	9	0	0.5742	0.0185	0.0119
OarFCB20	0.6754	0.6916	0.636	14	4	0.8144	0.1402	0.1172
SRCRSP7	0.2646	0.4247	0.3537	6	3	0.4767	0.1111	0.3171
ILSTS029	0.2508	0.3387	0.3003	10	1	0.3642	0.0856	0.4897
SPS113	0.6344	0.6832	0.626	15	3	0.7692	0.0988	0.4282
CSRD247	0.5538	0.661	0.6109	18	0	0.8515	0.2287	0.0000
MAF209	0.4365	0.4653	0.3508	2	0	0.4743	0.0287	0.0953
SRCRSP8	0.7134	0.7953	0.7482	19	0	0.8405	0.0472	0.0145
SRCRSP23	0.6928	0.8064	0.7680	22	1	0.8795	0.0712	0.1378
SRCRSP9	0.6158	0.7298	0.6825	13	1	0.7556	0.0393	0.0462
SRCRSP15	0.445	0.4673	0.4128	5	1	0.4886	0.0742	0.3688
TCRVB6	0.5895	0.6586	0.5979	15	0	0.7372	0.1009	0.3934
MAF70	0.3582	0.4592	0.4266	11	2	0.5605	0.2316	0.0301
OarFCB48	0.6369	0.6466	0.5903	12	2	0.6929	0.0686	0.3459
OarAE54	0.5332	0.6497	0.5983	16	1	0.7313	0.1018	0.3866
TGLA53	0.5443	0.5535	0.5072	10	3	0.5854	0.0548	0.1370
MEAN	0.5209	0.5994	0.5436	11.4	0	—	—	—

Note

dHWE, number of populations deviated from Hardy–Weinberg equilibrium; *N*_A_, the total number alleles of each marker in all populations; PIC, polymorphism information content.

Across markers, the *H*_E_ within the population ranged from 0.5834 ± 0.0456 in NLG to 0.6206 ± 0.0377 in ZTG. The *H*_o_ ranged from 0.4667 ± 0.0243 in WGG to 0.5793 ± 0.0230 in FQG. The *N*_A_ ranged from 4.80 ± 1.61 (WGG) and 4.80 ± 1.64 (FQG) to 6.20 ± 2.93 (YLG) (Table 4).

**Table 4:** Polymorphism measures for the seven Yunnan goat populations

Pop	*H*_o_(±*SD*)	*H*_E_(±*SD*)	*N*_A_(±*SD*)	*F*_IS_	*p*‐Value	dHWE
FQG	0.5793 ± 0.0230	0.5933 ± 0.0331	4.80 ± 1.64	0.024	0.2311	1
HGG	0.5783 ± 0.0243	0.6194 ± 0.0375	5.70 ± 1.92	0.068	0.0114	1
LHG	0.4914 ± 0.0218	0.5855 ± 0.0402	6.15 ± 2.74	0.163	0.0004	5
NLG	0.5081 ± 0.0213	0.5834 ± 0.0456	5.85 ± 2.70	0.131	0.0004	5
WGG	0.4667 ± 0.0243	0.5905 ± 0.0337	4.80 ± 1.61	0.214	0.0004	4
YLG	0.5080 ± 0.0205	0.6034 ± 0.0496	6.20 ± 2.93	0.160	0.0004	8
ZTG	0.5143 ± 0.0230	0.6206 ± 0.0377	5.90 ± 2.61	0.174	0.0004	4

Note

Pa, number of private allele; dHWE, number of populations deviated (*p* < 0.01) from Hardy–Weinberg equilibrium.

FQG, Fengqing hornless goat; HGG, Mile red‐bone goat; LHG, Longling goat; NLG, Ninglang black goat; WGG, Black‐bone goat, YLG, Yunling black goat; ZTG, Zhaotong goat.

For the HWE of each marker, the most extreme locus, ILSTS005, deviated from HWE in six populations and the locus OarFCB20 deviated from HWE in 4. Among the goat populations studied, YLG showed eight markers (ILSTS005, OarFCB20, SRCRSP7, SPS113, SRCRSP23, TCRVB6, MAF70, and OarAE54) that deviated from HWE, while FQG and HGG showed one marker (ILSTS005, and MAF70, respectively) that deviated from HWE (Supporting Information).

The *F*_IS_ within the population ranged from 0.024 (*p* = 0.2311) in FQG to 0.214 in WGG (*p* = 0.0004). No population generated a *p*‐value for inbreeding coefficients that was significantly different from zero, indicating an adjusted nominal level (5%) of 0.00036 based on 2,800 randomizations of the *p*‐value for *F*_IS_ (Table 4).

**Figure 1:**
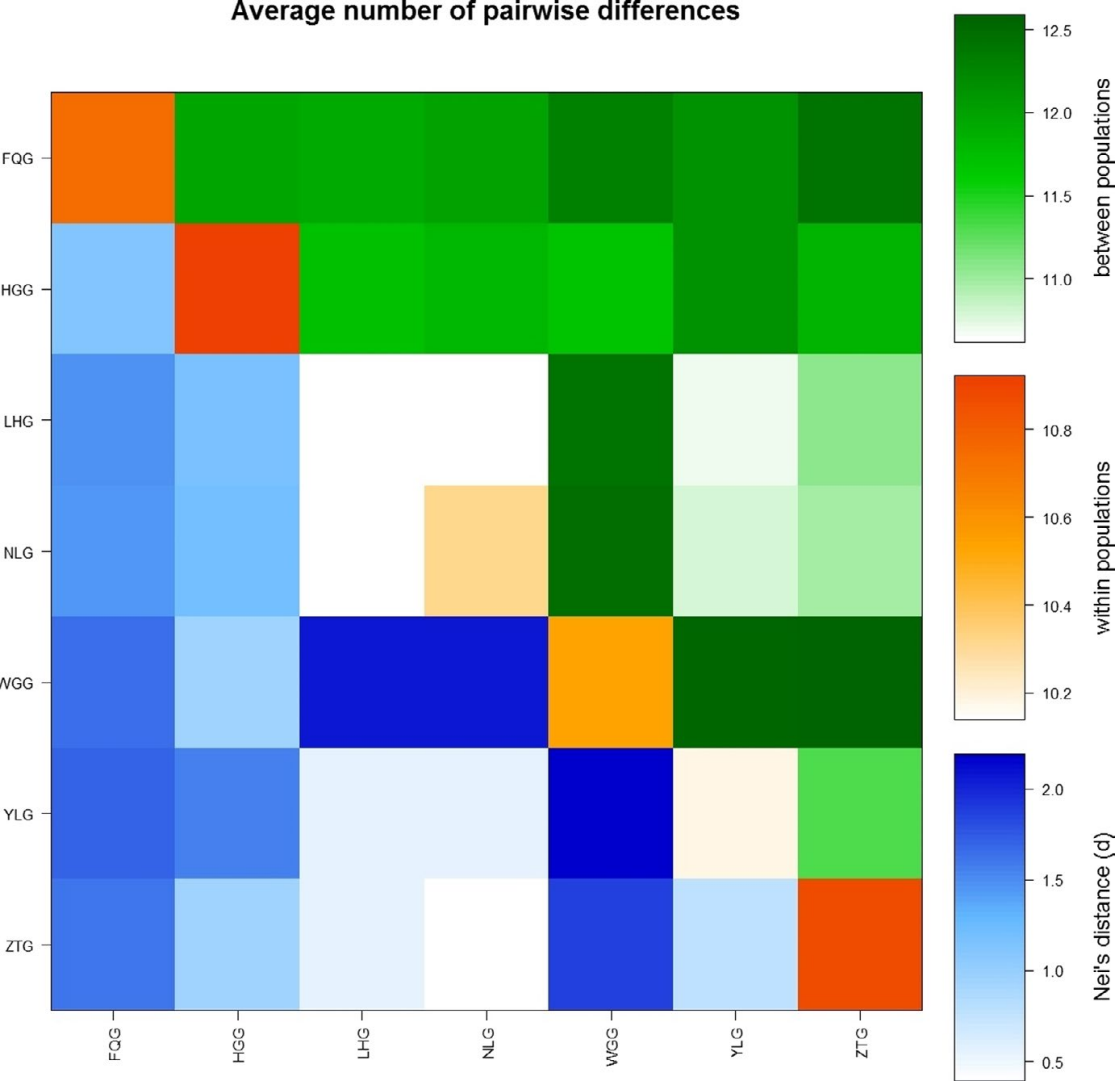
Population average pairwise differences among the seven goat populations. The area above the diagonal shows the average number of pairwise differences between populations, the diagonal elements represent the average number of pairwise differences within population, and the area below the diagonal shows the corrected average pairwise difference

In the *F*_ST_ analysis, the highest diversity within a population was observed for HGG (*πX* = 10.9233) and ZTG (*πX* = 10.8697), and the lowest diversity was observed for YLG (*πX* = 10.1867) and LHG (*πX* = 10.1377). YLG, ZTG, NLG, and LHG had the smallest differences between them compared with those between the other three populations (FQG, HGG, and WGG) when assessing the pairwise differences between populations (*πXY*), consistent with the results of the corrected average pairwise difference (*πXY*−[*πX* + *πY*]/2). In addition, according to the chi‐square test of *F*_ST_, all populations showed significant divergence (*p* < 0.05) from each other (Table 5, and Figure 1).

**Table 5:** Population average pairwise differences among the seven Yunnan goat populations

	FQG	HGG	LHG	NLG	WGG	YLG	ZTG
FQG	10.7488	11.9488^a^	11.9235^a^	11.9771^a^	12.2919^a^	12.1564^a^	12.4334^a^
HGG	1.1127^a^	10.9233	11.7108^a^	11.8125 a	11.6740^a^	12.1302^a^	11.8472^a^
LHG	1.4803^a^	1.1803^a^	10.1377	10.6184 a	12.4145^a^	10.7082^a^	11.0536^a^
NLG	1.4472^a^	1.1953 a	0.3940^a^	10.3110	12.4936^a^	10.7854^a^	10.9833^a^
WGG	1.6487 a	0.9435^a^	2.0768 a	2.0692^a^	10.5378	12.5568 a	12.5878 a
YLG	1.6887^a^	1.5752^a^	0.5460^a^	0.5366^a^	2.1946^a^	10.1867	11.3132^a^
ZTG	1.6242^a^	0.9507^a^	0.5500^a^	0.3929^a^	1.8841^a^	0.7850^a^	10.8697

Note

(1) Above diagonal: Average number of pairwise differences between populations (πXY); (2) Diagonal elements: Average number of pairwise differences within population (πX); (3) Below diagonal: Corrected average pairwise difference (*πXY* − (*πX* + *πY*).

FQG, Fengqing hornless goat; HGG, Mile red‐bone goat; LHG, Longling goat; NLG, Ninglang black goat; WGG, Black‐bone goat, YLG, Yunling black goat; ZTG, Zhaotong goat.

a The significant difference between populations (*p* < 0.05).

**Figure 2:**
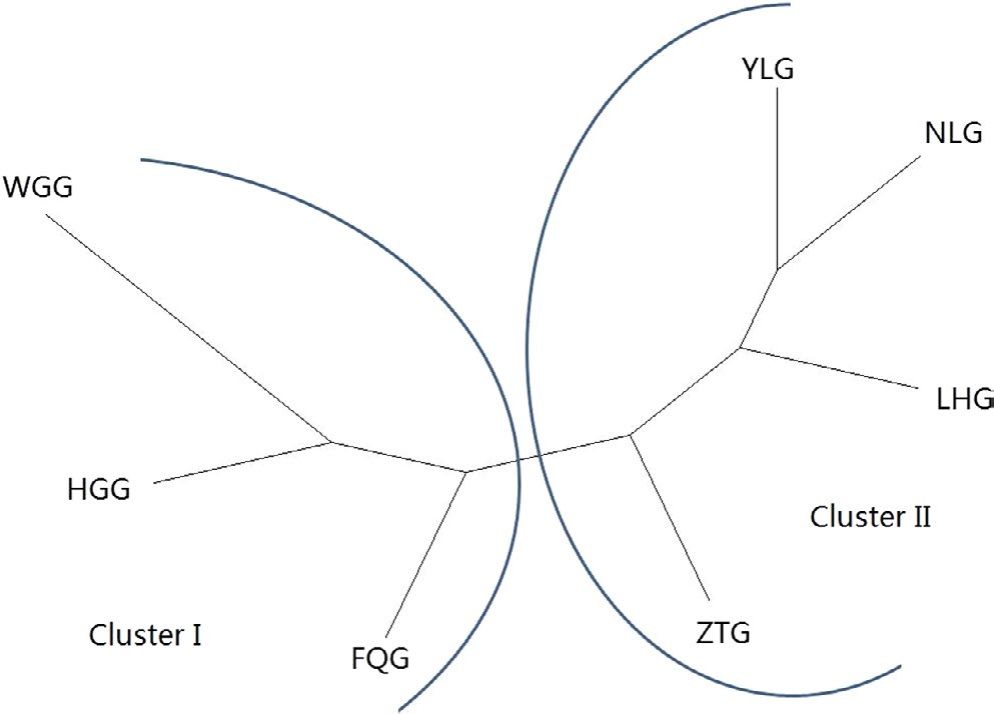
Neighbor‐joining network of the seven goat populations derived by Reynolds genetic distance by using five of the 20 genomic microsatellite markers

**Figure 3:**
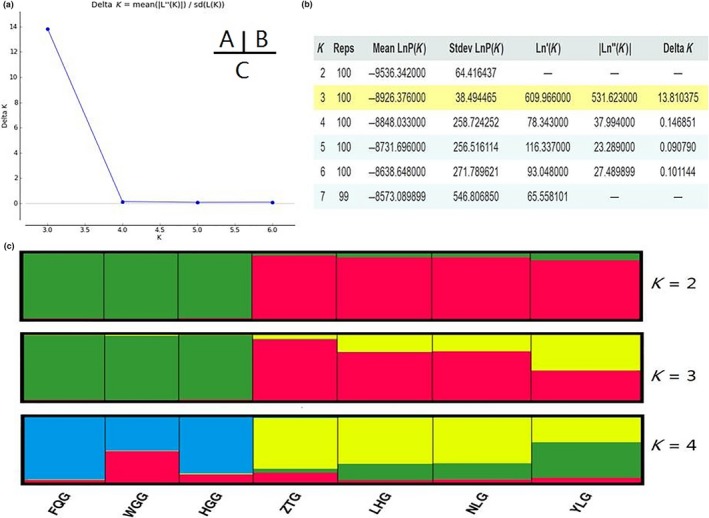
Clustering diagrams for the seven goat populations using STRUCTURE. The best *K* value is 3 according to Δ*K* = *m*| *L*″(*K*)|/*s*|*L*(*K*)|

**Figure 4:**
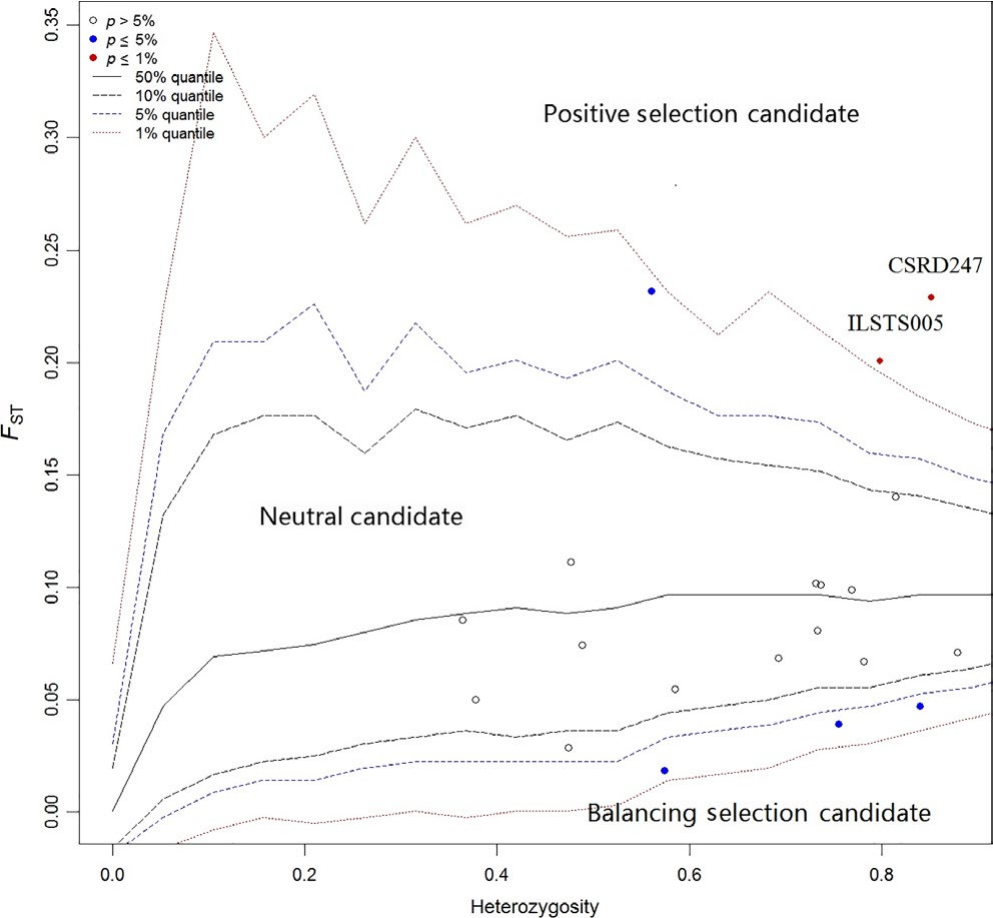
Detection of loci under selection from genome scans based on *F*_ST_

A phylogenetic neighbor‐joining network for the seven Yunnan goat populations was reconstructed (Figure 2). The FGQ, HGG, and WGG populations were separated into Cluster I, and the other four populations (YLG, ZTG, NLG, and LHG) were separated into Cluster II. STRUCTURE software was used for clustering individuals into 2 ≤ *K *≤ 10. The best *K* value was 3 by Δ*K* = *m*|*L*″ (*K*)|/*s*|*L*(*K*)|. From *K* = 2 to *K* = 4, seven studied populations were separated and formed two independent groups: group I, including FGQ, HGG, and WGG, and group II, including YLG, ZTG, NLG, and LHG (Figure 3). CSRD247 (*F*_ST_ *p*‐value = 0.0000) and ILSTS005 (*F*_ST_ *p*‐value = 0.0043) were found to be under positive selection in the locus selection style analysis (Figure 4).

## 4 DISCUSSION

In the present study, we assessed the genetic diversity and population structure of seven Yunnan indigenous goat populations using 20 microsatellites. The results showed high diversity (heterozygosis levels, PIC, and *N*_A_) of all microsatellite loci across the seven Yunnan goat populations, suggesting that these markers were adequate for assessing the diversity of these populations. In addition, several markers showed a deviation from HWE, which could have been due to the small sample size or the potential occurrence of recent population genetic events (E et al., 2018).

In a previous study, the diversity of three populations (YLG, ZTG, and FQG) analyzed in the present study was estimated, showing that the *H*_o_ was 0.5517, 0.5299, and 0.5178 in YLG, ZTG, and FQG, respectively (Wei et al., 2014), consistent with the results obtained in the present study, and indicating that the 20 highly polymorphic microsatellite loci utilized were sufficient to estimate the diversity of these seven populations.

In addition, the *H*_o_ value of every population was smaller than the *H*_E_ values, and 1–8 markers deviated from HWE within a population. Deviations from HWE occur when individual populations are substructured into smaller flocks within populations that are isolated from each other or improperly managed by humans due to inbreeding (E et al., 2018; Granevitze et al., 2007). In addition, the *F*_IS_ values for YLG (*F*_IS_ = 0.160) and ZTG (*F*_IS_ = 0.174) were higher than the values reported in a previous study (Wei et al., 2014), except for FQG. Although the *p* values for *F*_IS_ in the seven populations were not significantly different from zero after adjusting according to the nominal level (*p* = 0.00036), the *p* values for *F*_IS_ in five populations (LHG, NLG, WGG, YLG, and ZTG) were nearly significant (*p* = 0.0004). These findings further suggest that these populations might be more likely to experience inbreeding.

The pairwise *F*_ST_ observed from four populations (YLG, ZTG, NLG, and LHG) was generally lower than that observed between the other three breeds, indicating moderate to high genetic similarity among these breeds. The high genetic differences within the latter three populations compared to those within the four populations named above indicated a more complex genetic background of the domestication as well as less gene flow exchange between these breeds. This result was consistent with that obtained for phylogenetic network clustering.

The STRUCTURE analysis utilizing the Reynolds genetic distance revealed a clear clustering of these seven indigenous goats and was consistent with the results from the pairwise *F*_ST_ and phylogenetic network described above. Particularly, from *K* = 2 to *K* = 3, three populations (FQG, WGG, and HGG) were consistently separated, becoming an independent cluster. In addition, the other four goat breeds (ZYG, LHG, NLG, and YLG) were increasingly complex with an increasing *K*‐value reaching 4. A previous study reported that FQG showed the largest difference from Guishan goat and Maguan hornless goat (MGS) and the lowest genetic divergence from LHG and Yunling goat (YLG) using mtDNA variants (Wang, Hao, & Yang, 2016). Therefore, the present study provides supporting evidence indicating that a gene flow existed recently or during multicomplex ancient domestication of these four goats. Moreover, Wei et al. (2014) estimated the diversity through a PCA of forty Chinese goat populations by using 30 microsatellites, and the results indicated that FQG was not included in the Southwest goat group comprising HLG and MGS, which are indigenous to Yunnan (Wei et al., 2014). In the present study, we showed that this strange phenomenon not only involved FQG but also WGG and HGG.

Southwest China has complex river systems, a complex dynamic geographical and cultural history, and the richest biodiversity in China (Yuan, Cheng, & Zhou, 2012). The YLG samples were collected from Chuxiong, which is near Kunming and Panzhihua of Sichuan Province; the NLG samples were collected from the Ninglang region, which is also near Sichuan; and the LHG samples were collected from Longling, which is near Baoshan City, an area west of Yunnan. Notably, ZTG is an indigenous breed of the second largest city of Yunnan, Zhaotong, which is an important gateway to Yunnan and Guizhou Provinces. Zhaotong is an important channel for central plains culture to enter Yunnan and one of the three birthplaces of Yunnan culture in history. Thus, the geographic and cultural characteristics of these regions indicate a strong gene flow increase by human migration, commercial trade, and extensive transport (E et al., 2018; Zhao, Yu, Zhang, Kong, & Zhao, 2013).

However, WGG, FQG, and HGG samples, which were collected from the northwest vertical valley of the Hengduan Mountains, suggest that this geographic barrier may not only accelerate the exchange of genetic material in domestic animals but also reduce the frequency of communication with external gene flows. Until recently, increasing evidence has supported a common genetic pattern in domestic animals (Jin et al., 2012; Lai, Chen, Liu, & Yao, 2007; Liu et al., 2006; Wu et al., 2007) due to the restricted gene flow of livestock between goats as a result of the Hengduan Mountains (Wei et al., 2014).

Last, but not least important, we identified two markers (CSRD247 and ILSTS005) under positive selection. The domestic and wild animals were clustered according to their geographic locations and breeding and management histories by using microsatellites (Granevitze et al., 2007; Gvozdanović et al., 2018; Su et al., 2017). Those widely used microsatellite markers were codominant and assumed to be neutral drifting. However, positive selection by gradual fixation of dominant genotypes leads to a decline in genetic diversity in the population at this locus and an increase in population disagreement (Gordo et al., 2018; Liu et al., 2012; Rasigade, Hollandt, & Wirth, 2018). Therefore, we suggest that these two sites (CSRD247 and ILSTS005) under positive selection should be removed from the microsatellite evaluation system of goats to avoid interference from the positive selection sites on the population structure and genetic diversity evaluation. However, further studies are needed to determine whether this finding is appropriate.

## 5 CONCLUSION

In the present study, we estimated the diversity and population structure of 175 Yunnan goats from seven indigenous populations using 20 microsatellite markers. The results indicated that although these goats showed high diversity within the populations, the risk of inbreeding still existed. Second, the two‐population structures clustering from seven goats could be caused by geographical, economic, and cultural characteristics. Finally, we recommend removing CSRD247 and ILSTS005 loci from future studies on microsatellite evaluation system of goats, due to possible positive selection.

## CONFLICT OF INTEREST

None declared.

## AUTHOR CONTRIBUTIONS

YFH, GXE, and QHH, participated in the experimental design and wrote the manuscript; YJZ, MXC, YHM, LZ performed the laboratory experiment and carried out the bioinformatics data analysis; GXE have read and approved the final manuscript.

## Data Availability

We agree to deposit your data to a public repository, and the microsatellite genotypes: Dryad https://doi.org/10.5061/dryad.dk16pr0.
