# Supplementary material for: Genetic diversity estimation of Yunnan indigenous goat breeds using microsatellite markers
Source: Ecol Evol. 2019 Apr 29;9(10):5916–24. doi: 10.1002/ece3.5174 (PMC6540658; doi:10.1002/ece3.5174)
Supplement: Supplementary file 1 [file ECE3-9-5916-s001.docx]

Supplementary material I Hardy-Weinberg equilibrium of each marker within population

S1 Hardy-Weinberg equilibrium of each marker within FQG

| Marker | Observed heterozygosity | Expected heterozygosity | P-Value |
| --- | --- | --- | --- |
| ILSTS005 | 0.73913 | 0.66473 | 0.00773 |
| INRABERN185 | 0.3913 | 0.457 | 0.336 |
| MAF065 | 0.56522 | 0.657 | 0.39281 |
| INRA063 | 0.65217 | 0.65217 | 0.53552 |
| ILSTS011 | 0.73913 | 0.64831 | 0.98679 |
| OarFCB20 | 0.73913 | 0.64155 | 0.94023 |
| SRCRSP7 | 0.17391 | 0.34783 | 0.03553 |
| ILSTS029 | 0.52174 | 0.6058 | 0.2327 |
| SPS113 | 0.65217 | 0.52947 | 0.96503 |
| CSRD247 | 0.13043 | 0.47343 | 0 |
| MAF209 | 0.5 | 0.45983 | 1 |
| SRCRSP8 | 0.86957 | 0.75266 | 0.03488 |
| SRCRSP23 | 0.82609 | 0.80386 | 0.73575 |
| SRCRSP9 | 0.6087 | 0.73623 | 0.12276 |
| SRCRSP15 | 0.73913 | 0.69662 | 1 |
| TCRVB6 | 0.73913 | 0.64155 | 0.72343 |
| MAF70 | 0.21739 | 0.2058 | 1 |
| OarFCB48 | 0.52174 | 0.50435 | 0.41896 |
| OarAE54 | 0.47826 | 0.66957 | 0.02774 |
| TGLA53 | 0.78261 | 0.71787 | 0.3622 |

S2 Hardy-Weinberg equilibrium of each marker within HGG

| Marker | Observed heterozygosity | Expected heterozygosity | P-Value |
| --- | --- | --- | --- |
| ILSTS005 | 0.65 | 0.65513 | 0.93831 |
| INRABERN185 | 0.2381 | 0.22532 | 1 |
| MAF065 | 0.70588 | 0.76649 | 0.21684 |
| INRA063 | 0.61905 | 0.65621 | 0.7848 |
| ILSTS011 | 0.55 | 0.49359 | 0.83437 |
| OarFCB20 | 0.80952 | 0.66202 | 0.65781 |
| SRCRSP7 | 0.28571 | 0.49361 | 0.02114 |
| ILSTS029 | 0.33333 | 0.36121 | 0.66357 |
| SPS113 | 0.71429 | 0.63182 | 0.55745 |
| CSRD247 | 0.85 | 0.87436 | 0.12196 |
| MAF209 | 0.61905 | 0.50755 | 0.39266 |
| SRCRSP8 | 0.90476 | 0.82695 | 0.57785 |
| SRCRSP23 | 0.57143 | 0.82346 | 0.05941 |
| SRCRSP9 | 0.57143 | 0.75958 | 0.02737 |
| SRCRSP15 | 0.42857 | 0.56794 | 0.19185 |
| TCRVB6 | 0.61905 | 0.6144 | 0.94348 |
| MAF70 | 0.47619 | 0.77584 | 0 |
| OarFCB48 | 0.66667 | 0.6806 | 0.02575 |
| OarAE54 | 0.57143 | 0.59814 | 0.12345 |
| TGLA53 | 0.38095 | 0.41347 | 0.4722 |

S3 Hardy-Weinberg equilibrium of each marker within LHG

| Marker | Observed heterozygosity | Expected heterozygosity | P-Value |
| --- | --- | --- | --- |
| ILSTS005 | 0.25926 | 0.55695 | 0.0006 |
| INRABERN185 | 0.25926 | 0.32425 | 0.24791 |
| MAF065 | 0.59259 | 0.68553 | 0.47637 |
| INRA063 | 0.62963 | 0.66387 | 0.19639 |
| ILSTS011 | 0.33333 | 0.4102 | 0.21293 |
| OarFCB20 | 0.65385 | 0.78808 | 0.00003 |
| SRCRSP7 | 0.5 | 0.50798 | 1 |
| ILSTS029 | 0.22222 | 0.29769 | 0.02668 |
| SPS113 | 0.51852 | 0.77219 | 0.00034 |
| CSRD247 | 0.51852 | 0.62963 | 0.04022 |
| MAF209 | 0.29167 | 0.43883 | 0.15091 |
| SRCRSP8 | 0.66667 | 0.77918 | 0.01209 |
| SRCRSP23 | 0.85185 | 0.8805 | 0.19139 |
| SRCRSP9 | 0.65385 | 0.66742 | 0.93271 |
| SRCRSP15 | 0.25 | 0.4016 | 0.0955 |
| TCRVB6 | 0.62963 | 0.62963 | 0.2574 |
| MAF70 | 0.19231 | 0.25189 | 0.12602 |
| OarFCB48 | 0.62963 | 0.73585 | 0.00379 |
| OarAE54 | 0.59259 | 0.67156 | 0 |
| TGLA53 | 0.58333 | 0.61791 | 0.01391 |

S4 Hardy-Weinberg equilibrium of each marker within NLG

| Marker | Observed heterozygosity | Expected heterozygosity | P-Value |
| --- | --- | --- | --- |
| ILSTS005 | 0.28571 | 0.64221 | 0.00026 |
| INRABERN185 | 0.35714 | 0.47987 | 0.03512 |
| MAF065 | 0.57692 | 0.78431 | 0.02011 |
| INRA063 | 0.53846 | 0.70814 | 0.03245 |
| ILSTS011 | 0.57143 | 0.63766 | 0.37843 |
| OarFCB20 | 0.60714 | 0.68442 | 0.00001 |
| SRCRSP7 | 0.03571 | 0.13831 | 0.0002 |
| ILSTS029 | 0.25 | 0.33831 | 0.2033 |
| SPS113 | 0.42857 | 0.64286 | 0.00071 |
| CSRD247 | 0.46429 | 0.50649 | 0.27768 |
| MAF209 | 0.36 | 0.39265 | 0.64091 |
| SRCRSP8 | 0.85714 | 0.8513 | 0.48194 |
| SRCRSP23 | 0.82143 | 0.9 | 0.02607 |
| SRCRSP9 | 0.7037 | 0.69602 | 0.26217 |
| SRCRSP15 | 0.51852 | 0.45213 | 1 |
| TCRVB6 | 0.75 | 0.72403 | 0.14208 |
| MAF70 | 0.25 | 0.22662 | 1 |
| OarFCB48 | 0.67857 | 0.72208 | 0.44266 |
| OarAE54 | 0.57143 | 0.69805 | 0.0063 |
| TGLA53 | 0.53571 | 0.44221 | 0.89058 |

S5 Hardy-Weinberg equilibrium of each marker within WGG

| Marker | Observed heterozygosity | Expected heterozygosity | P-Value |
| --- | --- | --- | --- |
| ILSTS005 | 0.19048 | 0.59582 | 0.00005 |
| INRABERN185 | 0.42857 | 0.54355 | 0.20845 |
| MAF065 | 0.57143 | 0.76423 | 0.126 |
| INRA063 | 0.61905 | 0.58304 | 0.71242 |
| ILSTS011 | 0.33333 | 0.55285 | 0.0215 |
| OarFCB20 | 0.38095 | 0.55285 | 0.01447 |
| SRCRSP7 | 0.19048 | 0.48316 | 0.00755 |
| ILSTS029 | 0.09524 | 0.09408 | 1 |
| SPS113 | 0.61905 | 0.59466 | 0.93036 |
| CSRD247 | 0.57143 | 0.73635 | 0.04736 |
| MAF209 | 0.2381 | 0.50755 | 0.02533 |
| SRCRSP8 | 0.19048 | 0.63298 | 0 |
| SRCRSP23 | 0.61905 | 0.53542 | 0.61887 |
| SRCRSP9 | 0.33333 | 0.77468 | 0.00002 |
| SRCRSP15 | 0.61905 | 0.5122 | 0.33929 |
| TCRVB6 | 0.57143 | 0.58304 | 0.95716 |
| MAF70 | 0.80952 | 0.81649 | 0.30461 |
| OarFCB48 | 0.71429 | 0.69454 | 0.72266 |
| OarAE54 | 0.61905 | 0.65621 | 0.56436 |
| TGLA53 | 0.61905 | 0.59582 | 0.79067 |

S6 Hardy-Weinberg equilibrium of each marker within YLG

| Marker | Observed heterozygosity | Expected heterozygosity | P-Value |
| --- | --- | --- | --- |
| ILSTS005 | 0.32258 | 0.67478 | 0.00055 |
| INRABERN185 | 0.33333 | 0.29831 | 1 |
| MAF065 | 0.75862 | 0.73019 | 0.4664 |
| INRA063 | 0.58065 | 0.69963 | 0.27813 |
| ILSTS011 | 0.67742 | 0.66631 | 0.6993 |
| OarFCB20 | 0.62069 | 0.79068 | 0 |
| SRCRSP7 | 0.33333 | 0.57232 | 0.00352 |
| ILSTS029 | 0.16667 | 0.15763 | 1 |
| SPS113 | 0.8 | 0.83503 | 0.00894 |
| CSRD247 | 0.68966 | 0.75378 | 0.08689 |
| MAF209 | 0.48148 | 0.43955 | 1 |
| SRCRSP8 | 0.83871 | 0.844 | 0.07187 |
| SRCRSP23 | 0.46429 | 0.87273 | 0 |
| SRCRSP9 | 0.66667 | 0.71073 | 0.01615 |
| SRCRSP15 | 0.24138 | 0.22444 | 1 |
| TCRVB6 | 0.48387 | 0.68429 | 0.00254 |
| MAF70 | 0.10345 | 0.22202 | 0.00365 |
| OarFCB48 | 0.58065 | 0.54786 | 0.72033 |
| OarAE54 | 0.48276 | 0.77495 | 0 |
| TGLA53 | 0.53333 | 0.56836 | 0.28291 |

S7 Hardy-Weinberg equilibrium of each marker within ZTG

| Marker | Observed heterozygosity | Expected heterozygosity | P-Value |
| --- | --- | --- | --- |
| ILSTS005 | 0.08333 | 0.67465 | 0 |
| INRABERN185 | 0.125 | 0.19415 | 0.20486 |
| MAF065 | 0.78261 | 0.71884 | 0.98055 |
| INRA063 | 0.70833 | 0.73227 | 0.92535 |
| ILSTS011 | 0.54167 | 0.50621 | 1 |
| OarFCB20 | 0.91667 | 0.72163 | 0.00251 |
| SRCRSP7 | 0.33333 | 0.42996 | 0.27263 |
| ILSTS029 | 0.16667 | 0.51596 | 0.00014 |
| SPS113 | 0.70833 | 0.7766 | 0.19638 |
| CSRD247 | 0.65217 | 0.65314 | 0.37239 |
| MAF209 | 0.56522 | 0.51111 | 0.68966 |
| SRCRSP8 | 0.66667 | 0.88032 | 0.02764 |
| SRCRSP23 | 0.69565 | 0.82899 | 0.26772 |
| SRCRSP9 | 0.77273 | 0.76427 | 0.18953 |
| SRCRSP15 | 0.31818 | 0.41649 | 0.1456 |
| TCRVB6 | 0.33333 | 0.73316 | 0 |
| MAF70 | 0.45833 | 0.71543 | 0.01505 |
| OarFCB48 | 0.66667 | 0.64096 | 0.74298 |
| OarAE54 | 0.41667 | 0.47961 | 0.0944 |
| TGLA53 | 0.375 | 0.51862 | 0.02979 |
